# Severe caudal regression syndrome with overlapping features of VACTERL complex: antenatal detection and follow up

**DOI:** 10.1259/bjrcr.20150356

**Published:** 2016-12-08

**Authors:** K Kanagasabai, Venkatraman Bhat, GK Pramod, Siddaramappa J Patil, S Kiranmayi

**Affiliations:** ^1^Department of Imaging Services, Narayana Health, Mazumdar Shaw Cancer Center, Bengaluru, India; ^2^Department of Clinical Genetics, Mazumdar Shaw Cancer Center, Bengaluru, India; ^3^Department of Obstetrics, Mazumdar Shaw Cancer Center, Bengaluru, India

## Abstract

Caudal regression is a rare syndrome with a spectrum of structural defects involving multiple organ systems. Spinal anomalies, a charecteristic feature of the entity, can vary from isolated partial agenesis of the coccyx to lumbosacral agenesis with involvement of the thoracic spine in the most severe cases. The aetiology of this syndrome is not well-known. Maternal diabetes, genetic predisposition and vascular hypoperfusion have been suggested as possible causative factors. Severe forms of the disease are commonly associated with cardiac, renal and respiratory problems with overlapping feature of VACTERL complex (vertebral, anorectal, cardiac, tracheoesophageal, renal and limb anomalies). In this case report, we describe imaging appearances of severe caudal regression syndrome, VACTERL complex associated with multisystem anomalies, detected on a screening antenatal scan during second trimester. Some unusual features of the syndrome including sternal anomaly and absent bony hemithorax are highlighted.

## Clinical presentation

A 26-year-old female with prior bad obstetric history (G5 P5 A4 L0) was referred to the imaging service for a second trimester scan at an estimated gestational age of 22 weeks, which was her first scan in this present pregnancy. There was a history of consanguinity (uncle–niece union), three spontaneous abortions and one unexplained intrauterine death of a male foetus at 32 weeks gestation. She had a history of gestational diabetes mellitus in the immediately prior pregnancy. The laboratory evaluation did not reveal maternal hyperglycemia. Random blood sugar was 70 mg dl^−1^ (normal 70–140 mg dl^−1^) and HBA1c was 5.1% (normal ≤5.6 mg dl^−1^). Rest of the laboratory parameters were normal.

## Imaging findings

**Figure 1.**
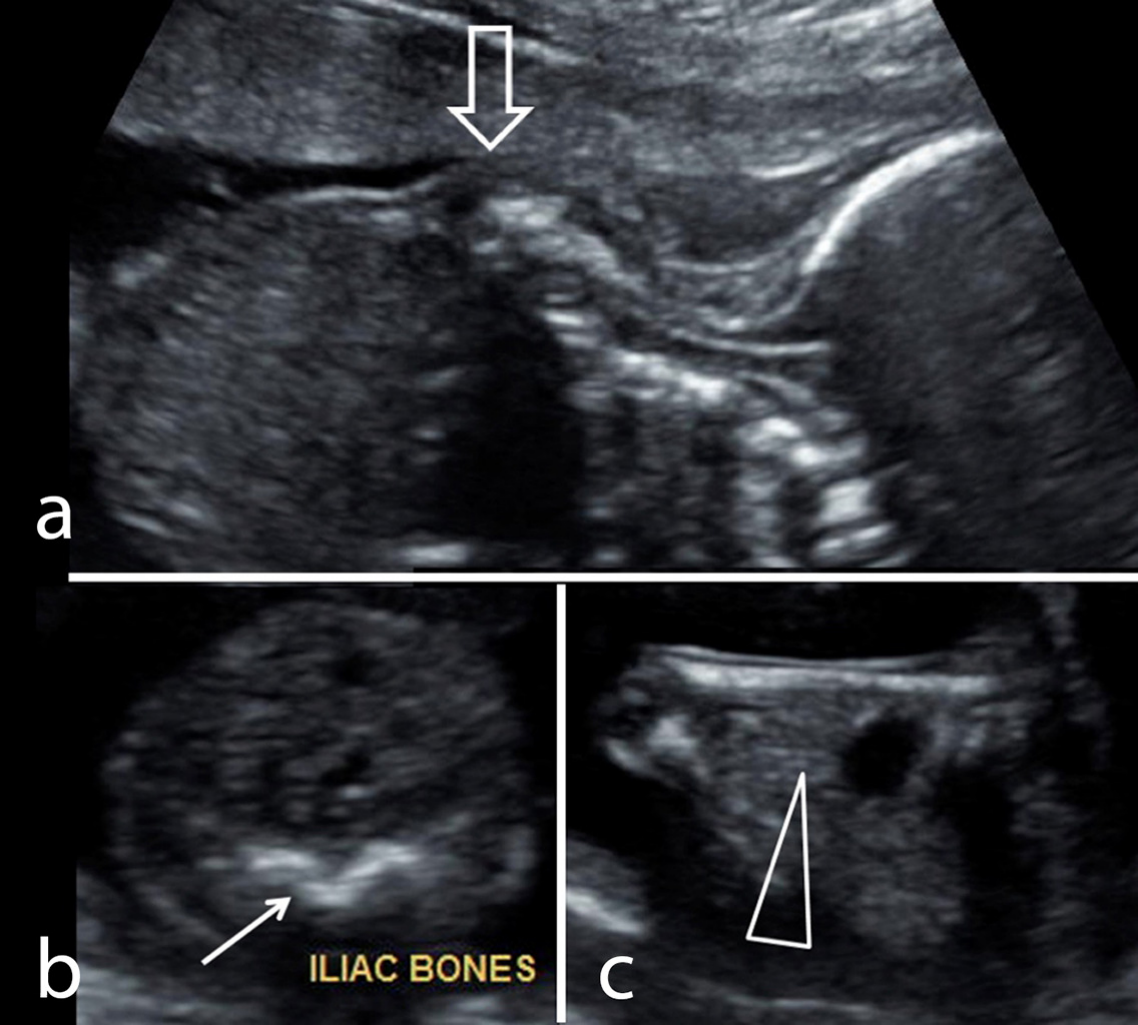
(a) Longitudinal sonogram of the foetus demonstrate a local hump in the dorsal region (open arrow) at the site of termination of the spinal column. No osseous elements are noted distally. (b) Transverse scan at the pelvic level shows fusion of both iliac bones in the midline, showing a “shield-like” configuration (arrow). (c) Image of the knee shows flexed position of the knee joint with a popliteal pterigium (arrow head). There is polyhydramnios.

**Figure 2.**
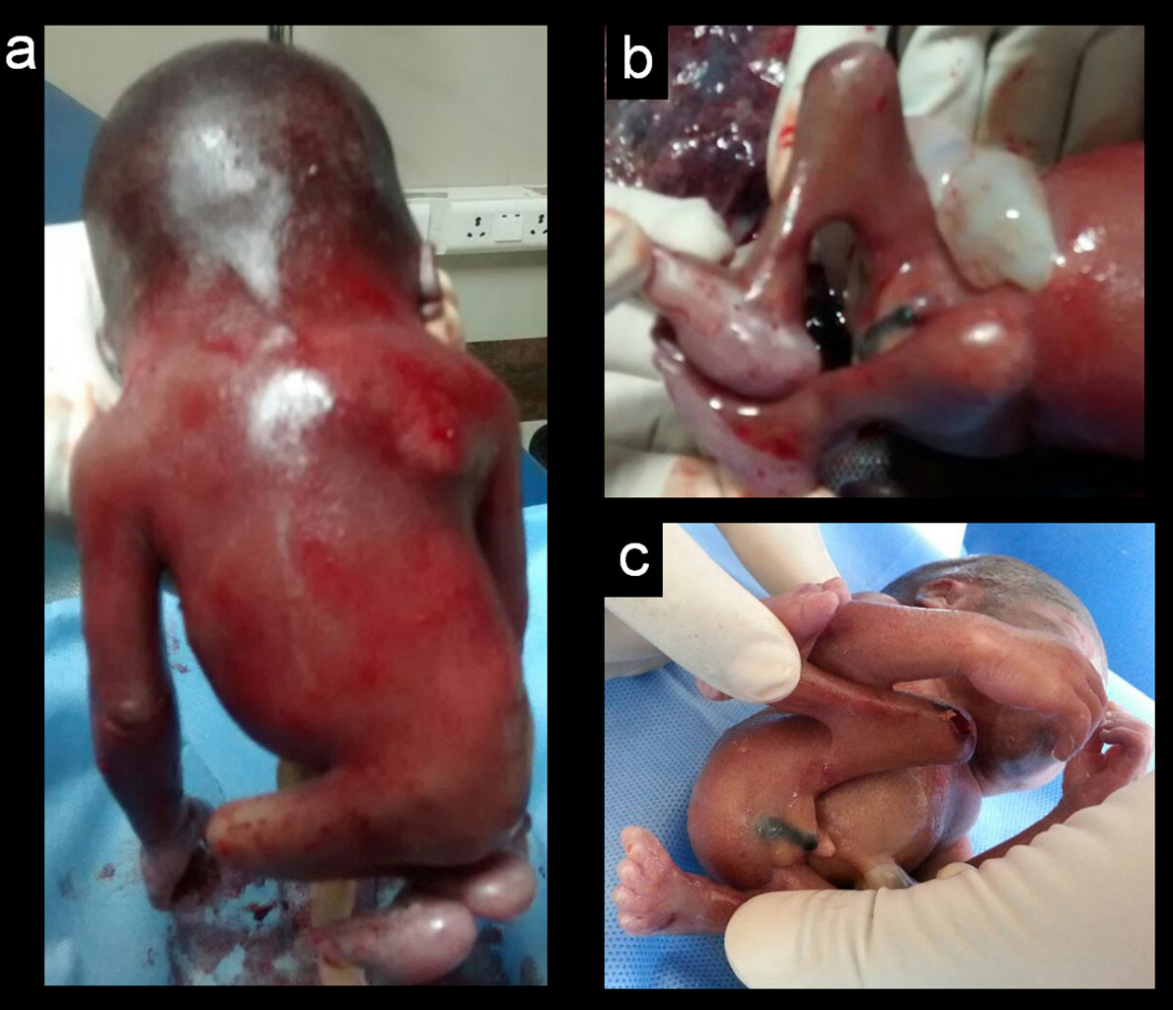
Aborted foetus viewed from behind (a) shows a short neck and the hump in the mid-dorsal region. Lower body parts are somewhat smaller (b) photograph demonstrating the flexion deformity of both knees with popliteal pterigium. There is also clubfoot deformity. (c) examination in the region of the perineum does not show anal dimple or orifice.

**Figure 3.**
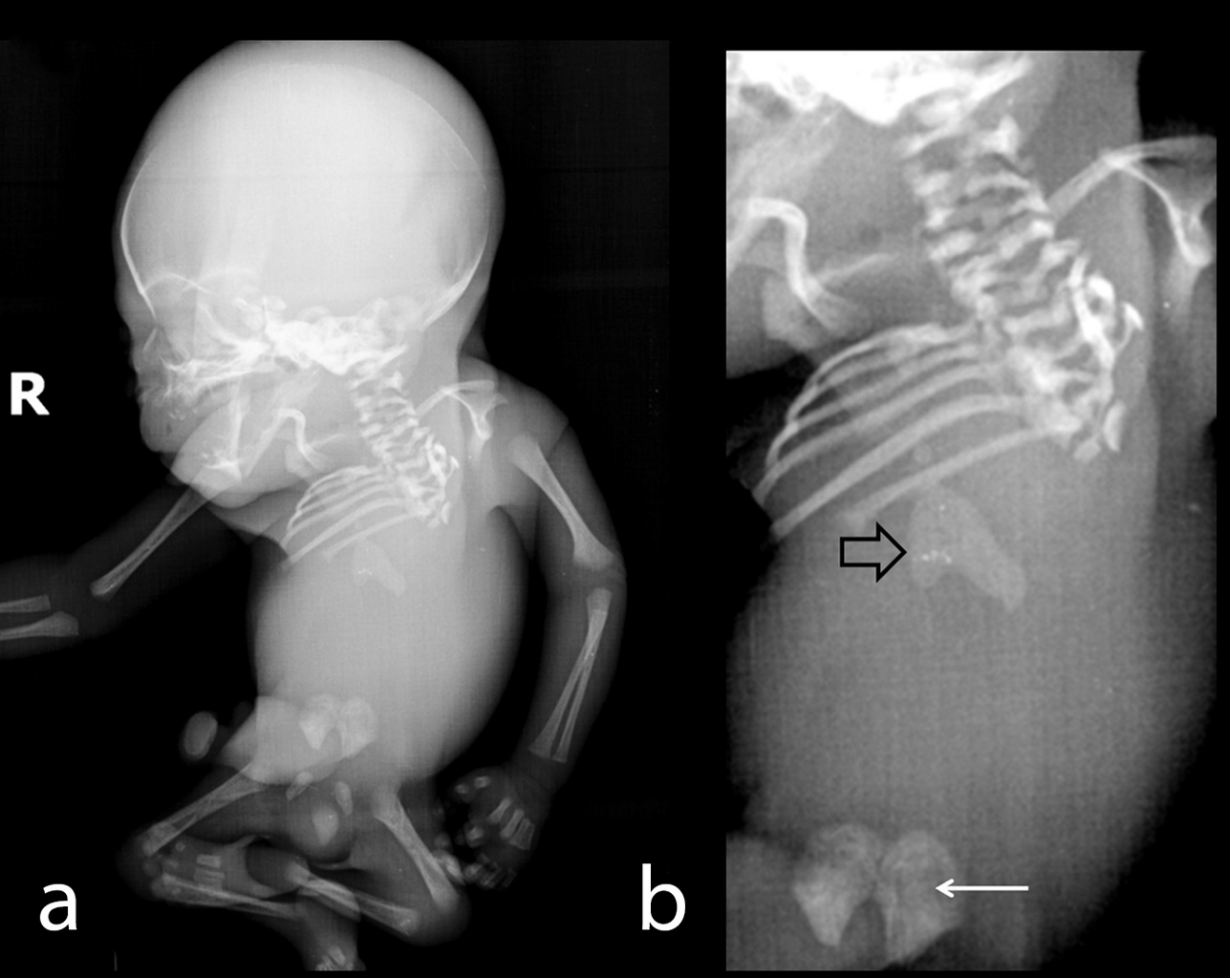
(a) Radiograph of the aborted foetus shows normal skull with the multiple spinal anomalies in cervical and dorsal region. Spinal structures show abrupt termination at the D7 vertebra. Left sided the ribs are absent. Both knee joints are flexed. Long bones of the upper and lower limbs appear normal (b) Close-up view demonstrates spinal anomalies and abrupt spinal termination. Lower sternum-xiphiod bony components shows hypertrophy and a bifid configuration (open arrow). Pelvic configuration is shown in the close-up view (arrow).

On ultrasound examination single intrauterine foetus was seen. There was polyhydramnios (amniotic fluid index: 21.5). The imaging of the of foetal spine revealed abrupt termination of the vertebral column at the distal thoracic level (D7–8) with abrupt cutoff of the spinal cord (Figure 1a). Lumbosacral spine was completely absent. Both iliac wings were fused in the midline, giving shield-like appearance (Figure 1b). The foetal lower extremities were akinetic with knee joints in persistent flexed position giving “frog leg” appearance. There was thin membrane in both popliteal fossa, representing popliteal pterygium (Figure 1c). Evaluation of foetal echocardiogram revealed cardiac levorotation, subaortic ventricular septal defectwith overriding of aorta and mild pulmonary stenosis. There was a single right kidney. Two-vessel cord was noted with atretic right umbilical artery. There was increased nuchal-fold thickness (13 mm). Intracranial appearance was normal. Findings were confirmed at autopsy (Figure 2) and radiography (Figure 3) of aborted foetus.

## Discussion

Caudal regression syndrome (CRS) is an uncommon malformation with incidence of approximately 0.01–0.05/1000 births, noted with higher frequency among diabetic mothers. CRS is characterized by complex vertebral anomalies in association with pelvic bone deformity. Frequently, there are anomalies of the lower limbs, gastrointestinal tract, genitourinary tract, heart and spinal cord.^1^

The embryological defect occurs at the mid-posterior axis of the mesoderm leading to arrest of progression of the mesoblastic caudal bud. Studies of axial mesoderm patterning at early gestation suggest that one or more processes of primitive streak migration, neuralisation or differentiation are compromised.^2^ It has been proposed that apoptosis, leading to derangement in cell proliferation is responsible for the development of caudal regression mediated through genetically defective dysfunctional telomeres.^3^ Some patients show additional features of VACTERL (vertebral, anorectal, cardiac, tracheoesophageal, renal and limb anomalies) along with typical presentation of CRS. While considering this complex entity, namely CRS-VACTERL association, at least three of six anomalies of VACTERL should be present.^4^ Based on position of conus, Pang^5^ divided patients into two groups: Group 1, in which conus ends cephalic to the lower border of L1 vertebrae. Patients of this category show large sacral deficit ending at or above S1 vertebrae. Group 2, in which conus ends caudal to the lower border of L1 vertebrae. Conus is elongated, tethered by thick filum terminale (65%). Other associations include terminal myelocystocoele (15%), terminal hydromyelia (10%) and terminal lipoma. Cardiac anomalies like ventricular septal defect are common in VACTERL association.^6^ Renal anomalies are unilateral renal agenesis, dysplastic–multicystic kidneys and duplication of collecting system.^7^ The preauricular tags seen in our case is a rare manifestation in CRS or VACTERL association with only a few reported cases.^8^ Popliteal pterigeum is a well-documented abnormality of the CRS, that can be detected on antenatal scans.

Classification of CRS helps to categorize patients from prognostic perspective.^5^ Renshaw classified the CRS into four types: Type I associated with total or partial or unilateral sacral agenesis, Type II showing partial sacral agenesis with bilaterally symmetrical defect, normal or hypoplastic sacral vertebra and a stable articulation between the ilia and first sacral vertebra, Type III with features of variable lumbar and total sacral agenesis with the ilia articulating with the sides of lowest vertebra and Type IV showing variable lumbar and total sacral agenesis, with caudal end plate of the lowest vertebra resting above either fused ilia. Salient features of Renshaw classification is highlighted in Table 1. Sirenomelia or “mermaid syndrome” charecterized by single femur and tibia with fusion of soft tissues of both the lower limbs is added as Type V.^9^ A prenatal sonographic diagnosis of CRS can be made conclusively with characteristic findings of abrupt interruption of the spine at the dorsal or lumbar level and abnormal leg position of the lower limbs. Femora show flexed “V” pattern, giving a typical “Buddha’s pose”. Ultrasound is very helpful in detecting associated-congenital malformations.^10,11^ Imperforate anus, although detectable, is often underdiagnosed on sonographic examination.^12,13^ There are reports of early diagnosis of CRS in the first trimester on transvaginal scan.^10^ Fukada et al reported a case of CRS on prenatal scan with increased nuchal translucency.^14^ Mild cases of CRS, especially Type I, shows some common features of Currarino syndrome which is charecterized by partial sacral agenesis, presacral mass and anorectal malformation.^15^

**Table 1.** Renshaw classification^9^ of causal regression syndrome

Type I	Partial or total unilateral sacral agenesis
Type II	Partial sacral agenesis with bilaterally symmetrical defect, normal or hypoplastic sacral vertebra and a stable articulation between the ilia and first sacral vertebra
Type III	Variable lumbar and total sacral agenesis with the ilia articulating with the sides of lowest vertebra present
Type IV	Variable lumbar and total sacral agenesis with the caudal endplate of the lowest vertebra resting above either fused ilia or an iliac amphiarthrosis

Our patient had normal glucose level in the current pregnancy, though had history of gestational diabetes. Several uncommon features were noted in our case: 1. Extent of spinal anomaly was very severe with spinal axis terminating at the level of the D7/8. 2. Foetus had agenesis of left-sided ribs, underdeveloped right hemithorax associated with bilateral pulmonary hypoplasia on foetal autopsy. 3. Foetus also had broad distal sternum with a bifid tip, a feature not previously described. Classifying our case into a precise category is difficult, as it shows overlapping features of the Renshaw IV and Pang Type I category. Although sonography provides vital information regarding the diagnosis of the syndrome, there may be a role for evaluation of the foetus either with MRI or low dose CT, to detect subtle additional abnormalities in soft tissues or osseous structures.

## Learning points

1. CRS with or without VACTERL association should be kept under serious consideration in high-risk patients, such as one with maternal diabetes and patients with positive family history
2. Sonography is a sensitive tool for the diagnosis and demonstration of classical manifestation of this syndrome. Familiarity with imaging appearance will facilitate detection and comprehensive evaluation.
3. Although the condition is frequently detected during second trimester there is potential for earlier diagnosis, allowing prompt management decision in severe cases.

## Consent

Publication consent as approved by institutional IRB.
